# Computational Recognition of a Regulatory T-cell-specific Signature With Potential Implications in Prognosis, Immunotherapy, and Therapeutic Resistance of Prostate Cancer

**DOI:** 10.3389/fimmu.2022.807840

**Published:** 2022-06-23

**Authors:** Mingyi Ju, Jingyi Fan, Yuanjiang Zou, Mingjie Yu, Longyang Jiang, Qian Wei, Jia Bi, Baohui Hu, Qiutong Guan, Xinyue Song, Mingyan Dong, Lin Wang, Lifeng Yu, Yan Wang, Hui Kang, Wei Xin, Lin Zhao

**Affiliations:** ^1^ Department of Pharmacology, School of Pharmacy, China Medical University, Shenyang, China; ^2^ Liaoning Key Laboratory of Molecular Targeted Anti-Tumor Drug Development and Evaluation, Liaoning Cancer Immune Peptide Drug Engineering Technology Research Center, Key Laboratory of Precision Diagnosis and Treatment of Gastrointestinal Tumors, Ministry of Education, China Medical University, Shenyang, China; ^3^ Department of Laboratory Medicine, The First Affiliated Hospital of China Medical University, Shenyang, China

**Keywords:** prostate cancer, regulatory T cells (Tregs), cancer immunotherapy (CI), therapeutic resistance, prognostic signature

## Abstract

Prostate cancer, recognized as a “cold” tumor, has an immunosuppressive microenvironment in which regulatory T cells (Tregs) usually play a major role. Therefore, identifying a prognostic signature of Tregs has promising benefits of improving survival of prostate cancer patients. However, the traditional methods of Treg quantification usually suffer from bias and variability. Transcriptional characteristics have recently been found to have a predictive power for the infiltration of Tregs. Thus, a novel machine learning-based computational framework has been presented using Tregs and 19 other immune cell types using 42 purified immune cell datasets from GEO to identify Treg-specific mRNAs, and a prognostic signature of Tregs (named “TILTregSig”) consisting of five mRNAs (*SOCS2, EGR1, RRM2, TPP1*, and *C11orf54*) was developed and validated to monitor the prognosis of prostate cancer using the TCGA and ICGC datasets. The TILTregSig showed a stronger predictive power for tumor immunity compared with tumor mutation burden and glycolytic activity, which have been reported as immune predictors. Further analyses indicate that the TILTregSig might influence tumor immunity mainly by mediating tumor-infiltrating Tregs and could be a powerful predictor for Tregs in prostate cancer. Moreover, the TILTregSig showed a promising potential for predicting cancer immunotherapy (CIT) response in five CIT response datasets and therapeutic resistance in the GSCALite dataset in multiple cancers. Our TILTregSig derived from PBMCs makes it possible to achieve a straightforward, noninvasive, and inexpensive detection assay for prostate cancer compared with the current histopathological examination that requires invasive tissue puncture, which lays the foundation for the future development of a panel of different molecules in peripheral blood comprising a biomarker of prostate cancer.

## Introduction

Prostate cancer is the most frequently diagnosed cancer and the second leading cause of cancer death in men worldwide, with an estimated incidence of 1,414,259 new cases in 2020, accounting for 7.3% of new cancer cases in men (1). Radical prostatectomy (RP) and radiation therapy (RT) are the most common primary treatment options for prostate cancer patients and can provide definitive cure in many patients. Unfortunately, recurrent prostate cancer following primary therapy is common (2), with the incidence of biochemical recurrence (BCR) ranging from 19% to 35% at 10 years following RP and approximately 30% following RT (3). Therefore, excavating a biomarker that can predict the recurrence holds the promise of improving survival for prostate cancer patients.

Recently, a growing body of evidence has revealed the attractive clinical efficacy of cancer immunotherapy (CIT) in the treatment of prostate cancer. For example, sipuleucel-T has been evaluated in the multicenter Immunotherapy for Prostate Adenocarcinoma Treatment (IMPACT) trial, which has been approved by the Federal Drug Administration, in addition to three Phase III clinical trials (NCT00065442, NCT00005947, and NCT01133704). Sipuleucel-T-treated patients tended to have a 3-fold increase in activated T cells in prostatectomy specimens compared to patients who did not receive sipuleucel-T (4). Furthermore, the median survival of patients who received sipuleucel-T was 25.8 months, while it was 21.7 months in placebo-treated patients. Beer et al. found that there was a significant difference seen in PFS between patients treated with ipilimumab and patients treated with placebo: 5.6 months in the ipilimumab group compared to 3.8 months in the placebo group (5). Despite the sustained clinical efficacy of CIT, however, only a fraction of patients benefit from them (6). Therefore, it has become a primary priority to excavate a biomarker that can accurately predict the prognosis and response to CIT for prostate cancer, which will bring tremendous value in guiding the management of prostate cancer patients. Previous studies have unveiled some indicators associated with CIT response such as tumor mutation burden (TMB) (7), eosinophilic count (8), PD-L1 expression (9), deep sequencing of T-cell receptor DNA (10), and glycolytic activity (11). However, accurate biomarkers for predicting clinical outcome and CIT responses for prostate cancer patients continue to be largely unexplored.

Prostate cancer, defined as a “cold” tumor, has an immunosuppressive microenvironment. Tumor-infiltrating lymphocytes (TILs) usually restrain the activity of T-effector cells, which may contribute to cancer progression. A previous study found that TILs residing in prostate cancer tissues were converted to regulatory T cells (Tregs) and T helper 17 (Th17) phenotypes, which suppressed autoreactive T cells and antitumor immune responses (12). In TILs, Tregs are numerous and highly activated (13, 14) and are supposed to represent a major mechanism of tumor-induced immune suppression (15, 16). Not only do Tregs inhibit T-effector cells, but they also appear to fundamentally alter the entire immune milieu related to the tumor. Overcoming the immunosuppressive tumor microenvironment (TME) is the major challenge impeding CIT today. Tregs are prevalent in nearly all cancers and, as immunosuppressive regulators of immune responses, they are the principal opponents of CIT (15, 17). Therefore, strategies to deplete Tregs and to control Tregs’ functions to increase anti-tumor immune responses are urgently required in the CIT field (18). In addition, Tregs have been observed to be significantly associated with poor prognosis of prostate cancer (19, 20). Transcriptional characteristics have recently been found to have a predictive power for the infiltration of Tregs, thus leading to the identification of gene expression biomarkers for quantitative evaluation of Tregs and prognosis and CIT response stratifications (21, 22). However, Treg-specific mRNAs and their application in evaluating Tregs and predicting prognosis and CIT responses have not been explored.

Though diagnosis of prostate cancer based on histopathological examination is accurate, this is not convenient for routine diagnosis because of the required invasive tissue puncture. Monitoring of peripheral blood is attractive for generating predictive biomarkers for prostate cancer, due to the ease of accessing blood versus tumor tissue. Blood is also more homogeneous compared to tumors, making the sampling of blood easier and more consistent. Moreover, for most patients who are candidates for CIT, the clinical utility of biomarkers is limited, and better ways to match patients with treatments are needed. Biomarkers isolated from peripheral blood are probably for drug screening and treatment monitoring. Therefore, due to the advantages of peripheral blood-based biomarkers, such as easy availability for analysis, the involvement of noninvasive procedures, the possibility for multiple assessments, and broad applications in diagnostics and monitoring therapeutic outcomes, there have been many studies dedicated to identifying predictive biomarkers using transcriptional profiles of peripheral blood mononuclear cells (PBMCs). For example, Zhou et al. identified an lncRNA signature of tumor-infiltrating B lymphocytes with potential implications in prognosis and immunotherapy of bladder cancer using transcriptional profiles of immune cells purified from PBMCs (23). Sun et al. also utilized transcriptional profiles of immune cells purified from PBMCs to develop a tumor immune infiltration-associated signature for non-small cell lung cancer (24).

Therefore, in this study, a machine learning-based computational framework is presented based on 42 transcriptional datasets of Tregs and other immune cell types purified from PBMC to identify Treg-specific mRNAs and develop a prognostic signature of Tregs (named “TILTregSig”) for monitoring prognosis of prostate cancer. The potential of the TILTregSig to serve as a predictive biomarker of CIT response and cancer therapeutic resistance was also explored.

## Methods

### Patient Data

Clinical information and transcriptional profiles of prostate cancer patients were retrieved from the Cancer Genome Atlas data portal (TCGA, http://cancergenome.nih.gov/). After the removal of patients with shorter than 1 month survival time from the data, 454 prostate cancer patients and their corresponding RNA sequencing (RNA-seq) data profiled expressed as transcripts per million (TPM) were obtained from the TCGA database. The R package “edgeR” was utilized to normalize and process the data by using the R version 4.0.4 software.

For validation, we also downloaded clinical information and transcriptional data of 25 prostate cancer patients (PRAD-FR cohort) from the International Cancer Genome Consortium (ICGC, https://dcc.icgc.org/).

We downloaded the immunotherapy response datasets from the GEO database (GSE19423, GSE111636, GSE67501, and GSE53922) and Miao et al. (25). The list of these immunotherapy response datasets is displayed in **Table S1**.

### Cell Culture

We obtained peripheral blood samples from 3 healthy donors and 3 prostate cancer patients. PBMCs were isolated by density gradient centrifugation with Ficoll-Paque (GE Healthcare). Human primary CD4+ CD25-T cells or CD4+ CD25+ Treg cells were purified using the CD4+ T Cell Isolation Kit (Miltenyi Biotec) and CD25 MicroBeads II (Miltenyi Biotec) (**Figure S1**). CD4+ CD25+ Treg cells were cultured with plate-bound anti-human-CD3 (OKT3; eBiosciences) antibodies (5 μg ml−1) and/or anti-human-CD28 (CD28.2; BD Pharmingen) antibodies (2 μg ml−1) in complete medium [RPMI supplemented with 10% FBS and IL-2 (Peprotech) (100 units ml−1)]. Then, CD4+ CD25+ CD127- Treg cells were enriched using flow cytometry (**Figure S1**). All cells were cultured at 37°C in an atmosphere of 5% CO2.

### Purified Immune Cell Data

Transcriptional profiles of T regulatory (Treg) cells and 19 other immune cell types conducted by GPL571 (Affymetrix Human Genome U133A 2.0 Array) were obtained from the publicly available GEO database (https://www.ncbi.nlm.nih.gov/geo/) including GSE38043, GSE11292, GSE22501, GSE22045, GSE23332, GSE65010, GSE43769, GSE50175, GSE42058, GSE59237, GSE23371, GSE37750, GSE14000, GSE4984, GSE27838, GSE8059, GSE46062, GSE17186, GSE39411, GSE50006, GSE13987, GSE85260, GSE56591, GSE16386, GSE16755, GSE13670, GSE11864, GSE84331, GSE51288, GSE44126, GSE26347, GSE142672, GSE134209, GSE101587, GSE66936, GSE49910, GSE28490, GSE93776, GSE67321, GSE72642, GSE28491, and GSE28726.

### Construction of a TILTreg-Derived mRNA Prognostic Signature for Prostate Cancer

**Figure 1:**
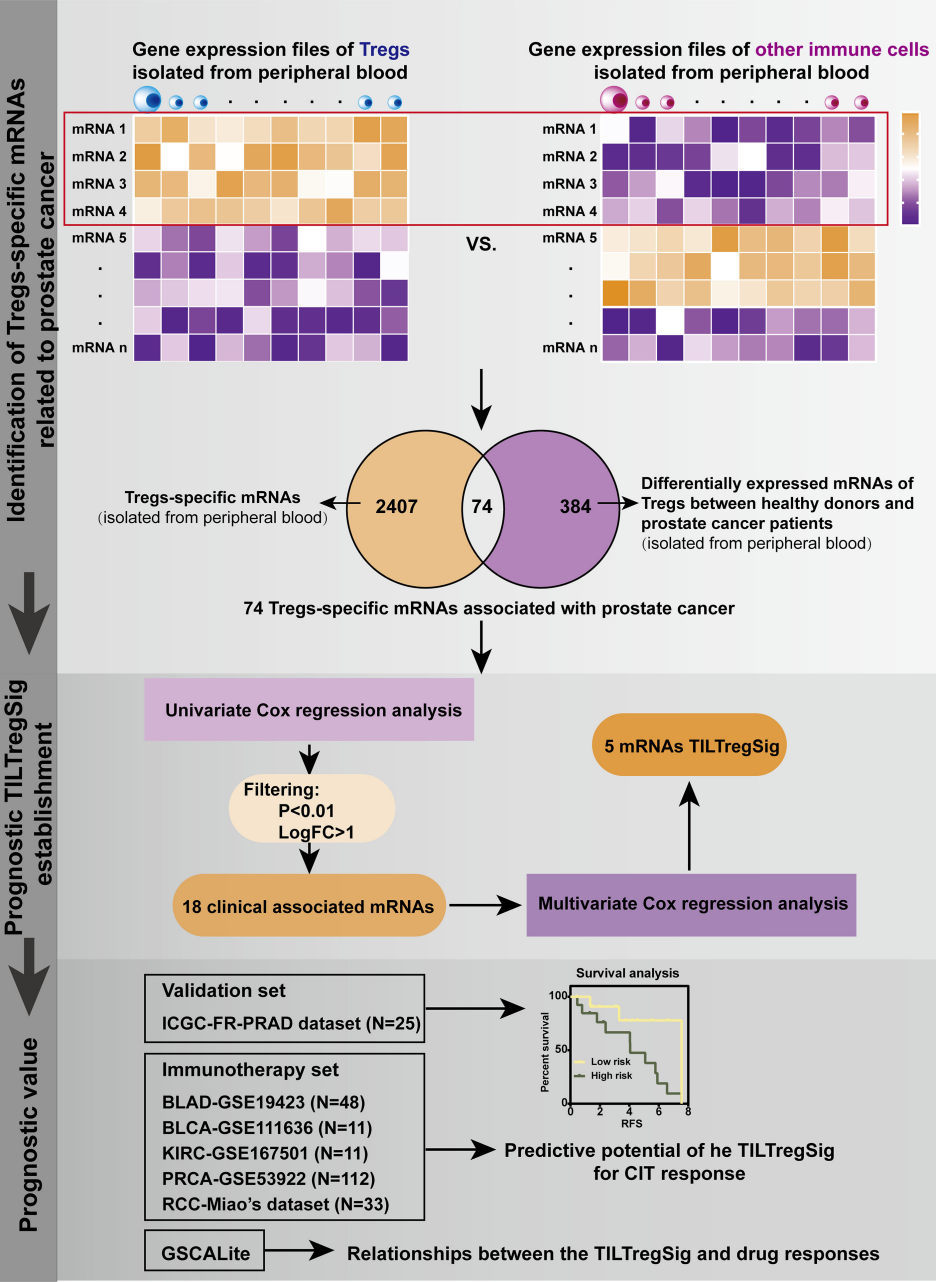
Schematic diagram of the framework for identification of the TILTregSig.

The mRNA and clinical profiling analysis was developed for identifying TILTregSig (i.e., the TIL-Treg-derived prognostic mRNA signature) as follows (**Figure 1**): (i) Differential expression analysis of mRNAs between Treg cell lines and other immune cell lines derived from PBMCs was performed using the R package “limma”. Those mRNAs highly expressed in Treg cell lines and downregulated in other immune cell lines were defined as Treg-specific mRNAs [by criterion as false discovery rate (FDR) < 0.05 and LogFC > 2]. (ii) To identify mRNAs that were associated with prostate cancer, a list of 384 genes differentially expressed between healthy donors and prostate cancer patients using gene expression profiles of Tregs excavated from PBMC of 3 healthy donors and 3 prostate cancer patients (26) was involved in this analysis. Treg-specific mRNAs that overlapped with these 384 genes were extracted out as Treg-specific mRNAs associated with prostate cancer (TILTreg-associated mRNAs). (iii) Univariate Cox regression analysis was used to explore TILTreg-associated mRNAs related to RFS as prognostic mRNAs. (iv) The machine learning approach was applied to select for the optimal combination from the list of candidate biomarkers for monitoring prognosis of prostate cancer (TILTregSig) based on multivariate Cox regression models and a forward and backward variable selection procedure *via* the “stepAIC” function from the R package “MASS”. The forward and backward variable selection model is one of the most basic and commonly used feature selection algorithms available and is also general and conceptually applicable to many different types of data (27). The combination of TILTreg-associated mRNAs related to the lowest Akaike information criteria (AIC) was retained as the final signature (named “TILTregSig”). (v) The corresponding risk scores for patients were calculated according to the expression levels of the genes (expi) and the coefficients of the multivariate Cox regression analysis (bi) for easy application in the clinic. Subsequently, patients were divided into low- and high-risk groups according to the mean risk score. The formula used was as follows:


$$\text{Riskscore}=\;\sum_{i=1}^{n}expi*bi$$

Malta et al. applied the one-class logistic regression (OCLR) machine-learning algorithm to TCGA datasets to calculate the stemness indices (mRNAsi and mDNAsi) using transcriptomic and epigenetic signatures (28). mDNAsi is reflective of epigenetic features, while mRNAsi is reflective of gene expression. Each stemness index (si) ranges from low (zero) to high (one) stemness.

### Cell Proliferation Assay

CD4+ CD25+ CD127- Treg cells were stimulated as described above and were then cultured with CD4+ CD25- T cells at a ratio of 1:1, 2:1, 4:1, and 8:1 (Teff:Treg) in each well of a round-bottom 96-well plate and cultured for 3 days. In the experimental group, the siGENOME SMARTpool and DharmaFECT 4 (Dharmacon) were used to knock down RRPM from CD4+ CD25+ CD127- Treg cells on a 96-well plate based on the manufacturer’s instruction. Si-RRPM Tregs were stimulated and were then cultured with CD4+ CD25- T cells as described above. The proliferation of the cells was monitored using Cell Counting Kit-8 (CCK-8) (Beyotime).

### Genetic Variation and Methylation Analysis of the TILTregSig

A webtool GSCALite (http://bioinfo.life.hust.edu.cn/web/GSCALite/) was used to analyze the genetic variation and methylation of the genes involved in the TILTregSig. Data in GSCALite overlapped with the samples derived from the TCGA database.

### Evaluation of Tumor Immunity, Tumor Mutation Burden, Glycolysis Score and Immune-Related Indicators, and Epithelial–Mesenchymal Transition Levels

In order to assess tumor immunity, we employed Estimation of STromal and Immune cells in MAlignant Tumors using Expression data (ESTIMATE), a method that quantifies the immune score, stromal score, ESTIMATE score, and tumor purity for each tumor sample as well as the immune activity (immune infiltration level) based on the expression of immune genes (29). For each tumor sample, we determined its TMB as the total count of somatic mutations (except silent mutations) detected in the tumor. Glycolysis score, an immune-related signature that has been proved by Jiang et al. (11), was also estimated in our study for each tumor sample using ssGSEA based on the glycolytic gene set (11) to compare the ability to predict immunity with the TILTregSig. The ssGSEA algorithm was utilized to calculate different immune-related indicators (including APC co-inhibition, APC co-stimulation, chemokine receptors, type I; IFN response and type II IFN response, anti-inflammatory cytokines, pro-inflammatory cytokines, MHC class I;, cytolytic activity, HLA, and TILs) using their feature genes (**Table S2**). We used epithelial–mesenchymal transition (EMT) markers including EMT1, EMT2, and EMT3, which were reported by Mariathasan et al. (30) to ssGSEA to evaluate the levels of EMT for prostate cancer patients in different groups.

### Evaluation of Immune Cell Infiltrations

To ensure the accuracy of our results, in this work, we utilized three methods to estimate the immune cell infiltrations in prostate cancer. In the first method, we employed ssGSEA to calculate the immune cell infiltration levels using immune cells’ marker genes (**Table S2**). The second method is CIBERSORT algorithm (31), which can infer the relative proportions of 22 types of infiltrating immune cells using gene expression profiles obtained from the TCGA database. In the last method, we employ the ImmuneCell AI database (http://bioinfo.life.hust.edu.cn/ImmuCellAI/#!/), which can evaluate the abundance of 24 immune cells, composed of 18 T-cell subtypes and 6 other immune cells: B cell, NK cell, monocyte cell, macrophage cell, neutrophil cell, and DC cell.

### Gene Set Enrichment Analysis

To explore the potential biological functions of the TILTregSig, we conducted Gene Set Enrichment Analysis (GSEA) based on the curated gene sets “c7.all.v7.4.symbles.gmt [immunologic signature]”. Normalized p-value < 0.05 was considered to be statistically significant.

### Statistical Analysis

The expression profiles of mRNAs from TCGA and GEO were shown as raw data, and each mRNA was normalized by log2 transformation for further analysis. The t-test p < 0.05 was utilized to determine the statistical significance. We calculated the correlation between two variables using the Spearman method. The threshold of p < 0.05 (Spearman’s correlation test) indicates the significance of correlation. Kaplan–Meier (K-M) survival curves and log-rank tests were used to compare the survival distribution between the high-risk and low-risk groups *via* GraphPad Prism version 7.0. To compare the predictive power of different genomic features, time-dependent receiver operating characteristic (ROC) curve analysis was performed using the R package “survivalROC”, and the area under the ROC curve (AUC) was used to assess the predictive performance of the genomic features. A webtool, GSCALite (http://bioinfo.life.hust.edu.cn/web/GSCALite/), was used to analyze the relationships between the IC50 data of different molecules and the genes’ expression levels in the TILTregSig. The Kruskal–Wallis test was used for comparisons among multiple groups. All the statistical analyses were performed in R version 4.0.4 with additional Bioconductor packages. A two-tailed p < 0.05 was considered statistically significant.

## Results

### Identification of Treg-Specific mRNAs Associated With RFS of Prostate Cancer

To identify Treg-specific mRNAs derived from PBMCs, differential expression analysis of mRNAs was performed between Tregs and other immune cell lines, and 2,407 dysregulated mRNAs were identified that were highly expressed in Tregs and downregulated in other immune cell lines [false discovery rate (FDR) <0.05, LogFC >2, **Table S3**]. These 2,407 dysregulated mRNAs were proposed as Treg-specific mRNAs. Ngar-Yee Huen et al. identified 384 genes of Tregs that were differentially expressed between healthy donors and prostate cancer patients using gene expression profiles excavated from PBMC of 3 healthy donors and 3 prostate cancer patients (26). To explore mRNAs of Tregs that were associated with prostate cancer, we further extracted 74 mRNAs out of these 2,407 mRNAs, which overlapped with the 384 differentially expressed genes (**Table S4**). A large body of evidence indicated that recurrent prostate cancer following primary therapy is common with a high incidence of BCR (2). Therefore, we further applied these 74 Treg-specific mRNAs derived from PBMC to the TCGA database to evaluate their association with recurrence-free survival (RFS) of prostate cancer based on univariate Cox regression analysis. Subsequently, 18 mRNAs were obtained that were significantly associated with RFS of prostate cancer patients (**Table S5**, p < 0.01). In these 18 mRNAs, 11 mRNAs were adverse indicators, while 7 mRNAs were protective indicators for prostate cancer, which were identified to be significantly associated with RFS and defined as candidate mRNAs of the prognostic signature for prostate cancer.

### Construction of a Prognostic Signature That Can Predict RFS of Prostate Cancer

To explore a prognostic signature for monitoring RFS of prostate cancer, multivariate Cox regression models were employed using gene expression profiles and clinical information of 454 patients with prostate cancer obtained from the TCGA database. Thus, the prognostic signature, named “TILTregSig”, was composed of five Treg-specific mRNAs (SOCS2, EGR1, RRM2, TPP1, and C11orf54). The risk score system was built as follows: risk score = (−0.332 × expression value of SOCS2) + (−0.111 × expression value of EGR1) + (0.286 × expression value of RRM2) + (−0.609 × expression value of TPP1) + (−0.748 × expression value of C11orf54). A total of 454 PRAD patients were dichotomized into high- and low-risk groups according to the cutoff value of 0.946 as the median value of risk score.

**Figure 2:**
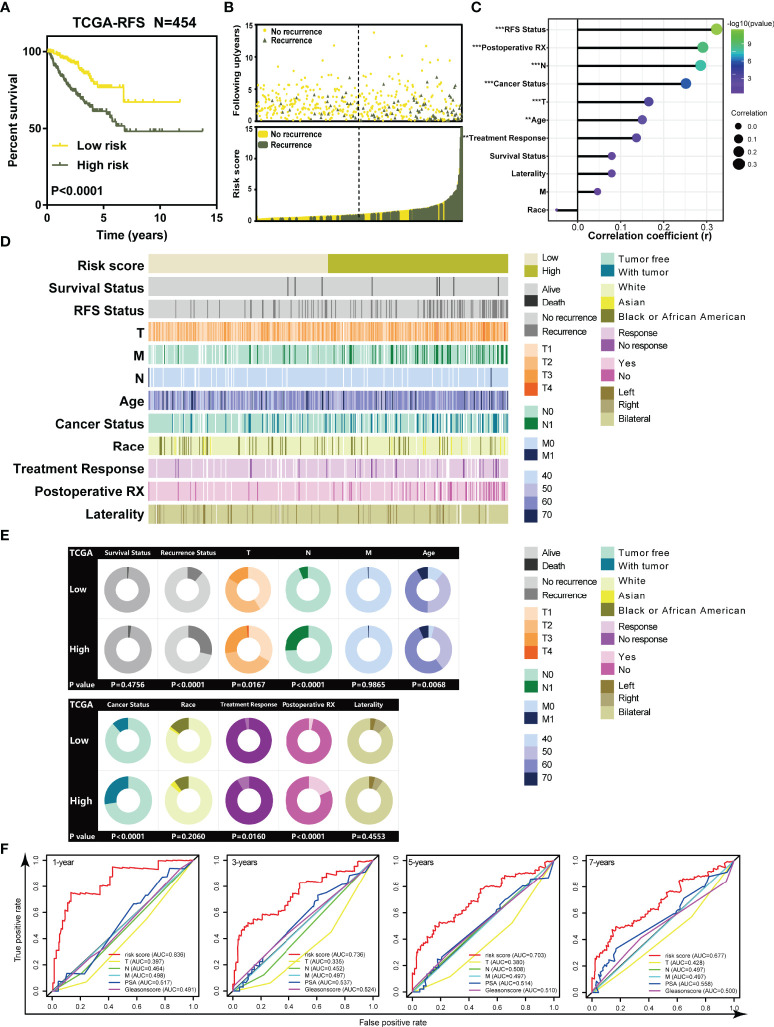
The TILTregSig can serve as a biomarker for RFS of prostate cancer patients in both TCGA and ICGA datasets. **(A)** Kaplan–Meier curves of RFS of low- and high-risk groups stratified by the TILTregSig in prostate cancer patients in the TCGA dataset. **(B)** The distribution of gene risk scores and patients’ recurrence status for prostate cancer patients in the TCGA dataset. **(C)** Correlation analysis between risk score and clinical characteristics in prostate cancer. *R*: Spearman’s correlation coefficient. **(D)** The strip chart of risk score and clinical characteristics for patients with prostate cancer in the TCGA dataset. **(E)** Pie charts showing the chi-square test of clinicopathologic factors for low- and high-risk groups in prostate cancer samples from the TCGA dataset. **(F)** Comparison of predictive ability of TILTregSig, PSA levels, Gleason score, Clinical T, Clinical N, and Clinical M for RFS of prostate cancer in 1, 3, 5, and 7 years using ROC curve analysis. **, P<0.01. ***, P<0.001.

Kaplan–Meier (K-M) curves revealed that patients in high-risk groups tended to suffer from recurrence (Log-rank p < 0.0001, **Figure 2A**). Patients’ recurrence rate was increased in the high-risk group compared to that in the low-risk group (**Figure 2B**). Correlation analysis indicated that RFS status, T, N, age, cancer status, treatment response, and postoperative RX were significantly associated with the risk score (**Figure 2C**, p < 0.05). The strip chart showed that the risk of patient mortality and recurrence rate obviously increased and the TNM staging gradually rose as the risk score increased (**Figure 2D**). The chi-square test demonstrated that the high-risk group significantly tended to recurrence (p < 0.0001, **Figure 2E**), higher T stage (p = 0.0167, **Figure 2E**), higher N stage (p < 0.0001, **Figure 2E**), being older (p = 0.0068, **Figure 2E**), survival with tumor (p < 0.0001, **Figure 2E**), no response to treatment (p = 0.0160, **Figure 2E**), and no postoperative RX (p < 0.0001, **Figure 2E**) compared to the low-risk group of prostate cancer. These results suggested that highly malignant prostate cancer was associated with high-risk score, and our risk score system based on the TILTregSig had tremendous potential to predict RFS for prostate cancer patients.

To better evaluate the predictive power of our TILTregSig for prostate cancer patients, we involved PSA levels, Gleason score, Clinical T, Clinical N, and Clinical M into this comparison analysis based on the available clinical information from the TCGA database. ROC curves indicated that the risk score based on our TILTregSig had moderate potential as a predictor of RFS in prostate cancer (**Figure 2F**). PSA levels and Gleason score presented low potential for predicting RFS, while Clinical T, Clinical N, and Clinical M showed minimal potential in prostate cancer (**Figure 2F**). These results suggest that the risk score based on our TILTregSig is a predictor with stronger power for RFS than PSA levels, Gleason score, Clinical T, Clinical N, and Clinical M as classically clinical parameters.

Cancer stem cells were proved to be associated with poor prognosis (32). Malta et al. employed the OCLR machine-learning algorithm to TCGA datasets to calculate the stemness indices (mRNAsi and mDNAsi) using transcriptomic and epigenetic signatures (28). Thus, we also involved stemness indices derived from study of Malta et al. to validate the clinical association between the TILTregSig and the prognosis of prostate cancer patients. The results showed a significantly positive correlation between the TILTregSig and the stemness indices in both the mRNA expression levels (p = 0.0006, R = 0.160, **Figure S2A**) and DNA methylation levels (p < 0.0001, R = 0.296, **Figure S2B**), which was consistent with our previous results where a high-risk score based on the TILTregSig was markedly associated with recurrence of prostate cancer.

### Validation of the TILTregSig in the ICGC Database and Experimental Data

**Figure 3:**
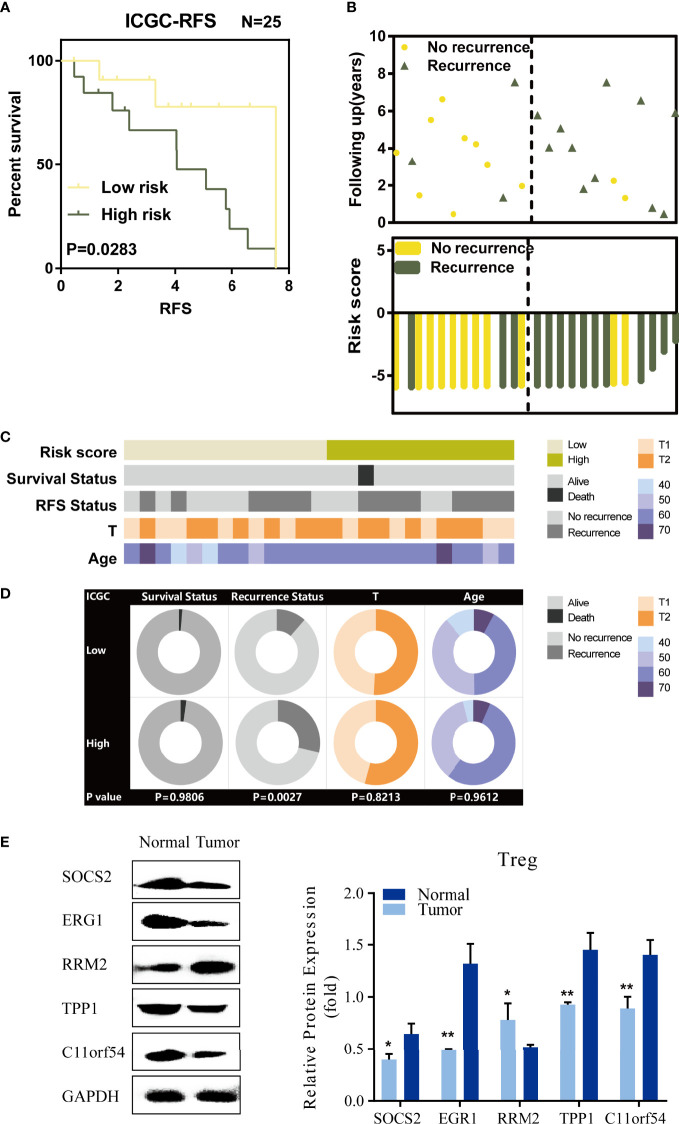
Validation of TILTregSig in the ICGC database and experimental data. **(A)** Kaplan–Meier curves of RFS of low- and high-risk groups stratified by the TILTregSig in prostate cancer patients in the ICGC dataset. **(B)** The distribution of gene risk scores and patients’ recurrence status for prostate cancer patients in the ICGC dataset. **(C)** The strip chart of risk score and clinical characteristics for patients with prostate cancer in the ICGC dataset. **(D)** Pie charts showing the chi-square test of clinicopathologic factors for low- and high-risk groups in prostate cancer samples from the ICGC dataset. **(E)** Comparison of the expression of five genes (SOCS2, EGR1, RRM2, TPP1, and C11orf54) based on our TILTregSig between Tregs excavated from PBMC of 3 healthy donors and 3 prostate cancer patients using Western blot analysis. *, P<0.05. **, P<0.01.

To validate the prediction power of the TILTregSig, we further applied the signature to ICGC database. Then, 25 prostate cancer patients were divided into low- and high-risk groups according to the median value of the risk score in the ICGC dataset. As shown in **Figure 3A**, K-M curves showed significant utility in predicting RFS of prostate cancer. We also found that patients’ recurrence rate was increased in the high-risk group compared to that in the low-risk group (**Figure 3B**). The strip chart of clinical characteristics of prostate cancer patients indicated that the risk of mortality and recurrence rate increased as the risk score increased (**Figure 3C**). The chi-square test showed that patients in the high-risk group markedly tended to suffer from recurrence (p = 0.0027, **Figure 3D**). These results illustrate that high-risk score is associated with recurrence of prostate cancer patients, which is consistent with our previous results in the TCGA dataset.

To validate the different expression levels of the five genes (SOCS2, EGR1, RRM2, TPP1, and C11orf54) based on our TILTregSig in Tregs between healthy donors and prostate cancer patients, we obtained Tregs excavated from PBMCs of 3 healthy donors and 3 prostate cancer patients, and carried out Western blot analysis. The results show in **Figure 3E** that SOCS2, EGR1, TPP1, and C11orf54 had significant lower expression in Tregs excavated from prostate cancer patients compared to those excavated from healthy donors, while RRM2 was significantly overexpressed in prostate cancer (**Figure 3E**), suggesting that RRM2 was a risk factor in our TILTregSig for prostate cancer patients. These results were consistent with our previous findings using bioinformatics analysis.

### The TILTregSig Is an Independently Prognostic Indicator for Prostate Cancer Patients

Next, univariate and multivariate Cox regression analysis was carried out to analyze whether the TILTregSig can be an independent predictor for prostate cancer patients. The risk score and other clinicopathological factors were used as covariates. Stage M was not included in this analysis because of only one patient in stage M1 in the TCGA dataset. The results unveiled that the risk score (Multivariate Cox: HR = 1.170, 95% CI = 1.079–1.267; p < 0.001), stage T (Multivariate Cox: HR = 1.749, 95% CI = 1.235–2.474; p = 0.002), and cancer status (Multivariate Cox: HR = 8.084, 95% CI = 4.557–14.33; p < 0.001) were significantly associated with the RFS and could be independent RFS prognostic factors for prostate cancer patients (**Figures S3A, B**).

To further investigate the clinical potentiality of the risk score in prostate cancer, stratified analysis based on these clinical characteristics was implemented. The results indicated that the TILTregSig seemed to be more applicable to predict RFS of prostate cancer patients in the subgroups of T1 and T2, N0, younger than 71, White, response to treatment, did not receive postoperative RX, and laterality in bilateral (**Figure S3C**). Meanwhile, patients in the high-risk group had significantly poorer clinical outcomes compared to those in the low-risk group. These results illustrate that the TILTregSig can serve as an independent predictor for RFS of prostate cancer patients, and still applicable for patients in some subgroups.

### The Landscape of Genetic Variations of the TILTregSig in Prostate Cancer

**Figure 4:**
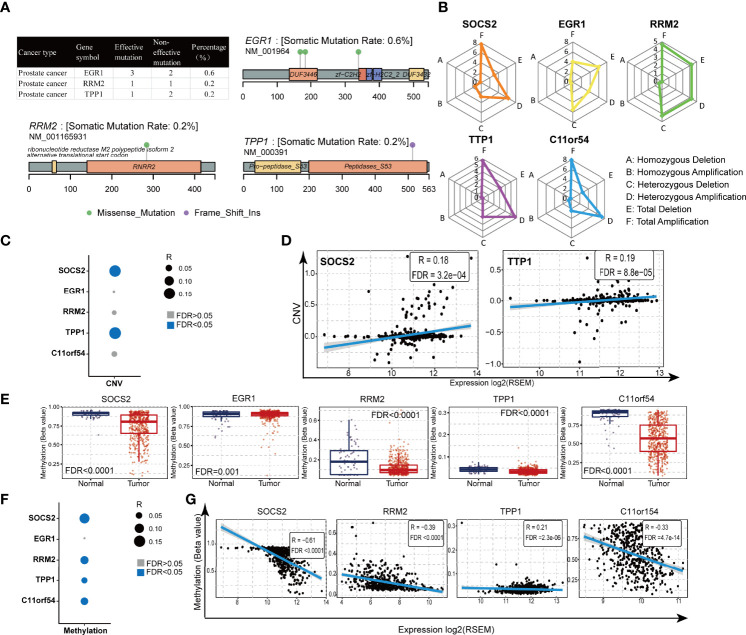
Gene alteration and methylation landscape of the five genes in the TILTregSig in prostate cancer. **(A, B)** The SNV status of the five genes in the TILTregSig of prostate cancer patients in GSCALite. **(C)** The correlation between CNV and the expression levels of the five genes in the TILTregSig of prostate cancer patients in GSCALite. **(D)** The correlation between CNV and the expression levels of SOCS2 and TPP1. *R*: Spearman’s correlation coefficient. FDR, false discovery rate. **(E)** The difference of gene methylation between normal and prostate cancer samples. **(F)** The correlation between gene methylation and the expression levels of the five genes in the TILTregSig of prostate cancer patients in GSCALite. **(G)** The correlation between gene methylation and the expression levels of SOCS2, RRM2, TPP1, and C11orf54. *R*: Spearman’s correlation coefficient. FDR, false discovery rate.

Genetic alterations have been found to usually confer susceptibilities to prostate cancer (33). GSCALite (http://bioinfo.life.hust.edu.cn/web/GSCALite/) is a webtool that can be used to analyze the genetic variation of the genes, and data in the GSCALite overlapped with the samples derived from the TCGA database. Therefore, in order to comprehensively understand the molecular characteristics of the five genes in the TILTregSig, we examined the SNV and CNV status of these genes using GSCALite. It was found that the EGR1 (0.6%) exhibited the highest mutation frequency followed by RRM2 (0.2%) and TPP1 (0.2%), while both SOCS2 and C11or54 did not show any mutations in prostate cancer samples (**Figure 4A**). In addition, EGR1 had three effective mutation sites, while both RRM2 and TPP1 had one site, respectively (**Figure 4A**). Among alteration types, most were focused on the amplification (SOCS2: 7.72%, RRM2: 4.67%, TPP1: 5.69%, and C11orf54: 7.93%, **Figure 4B**; **Table S6**), while EGR1 had a widespread frequency of deletion (EGR1: 6.1%, **Figure 4B**; **Table S6**). The investigation of the correlation between CNV and the expression levels of the five genes indicated a significant positive correlation of SOCS2 (**Figures 4C, D**; Spearman coefficient: R = 0.18, p = 3.2e−04) and TPP1 (**Figures 4C, D**; Spearman coefficient: R = 0.19, P = 8.8e−05) expression with CNV, which indicated that patients with high expression of SOCS2 and TPP1 were prone to have high CNV load. The above analysis presents a widespread genetic alteration landscape of the five genes in the TILTregSig in prostate cancer patients, suggesting genetic alterations as the molecular mechanism that shows that high-risk score is related to poor prognosis in prostate cancer.

### The Five mRNAs Involved in the TILTregSig Have Significant Differential Methylation Between Normal and Tumor Samples in Prostate Cancer

Gene methylation plays a vital role in malignant transformation and can be specific to types of cancers including prostate cancer (34). To get a better understanding of the mechanism of the effect of the genes in the TILTregSig on tumorigenesis, we analyzed the differential methylation of the five genes using GSCALite. Surprisingly, we found that all of the five genes showed significant differential methylation between normal and prostate cancer samples (p < 0.05, **Figure 4E**). Furthermore, SOCS2 (**Figures 4F, G**; Spearman coefficient: R = −0.61, p < 0.0001), RRM2 (**Figures 4F, G**; Spearman coefficient: R = −0.39, p < 0.0001), TPP1 (**Figures 4F, G**; Spearman coefficient: R = −0.21, p = 2.3e−06), and C11orf54 (**Figures 4F, G**; Spearman coefficient: R = −0.33, p = 4.7e−14) showed significant negative correlation between gene methylation and expression in prostate cancer, whereas the expression of EGFR (**Figure 4F**; p > 0.05) showed no significant correlation with gene methylation. These findings could contribute to enhancing our understanding of the potential mechanisms on the predictive ability of the TILTregSig in prostate cancer.

### The TILTregSig Is a Stronger Predictor for Tumor Immunity in Prostate Cancer

**Figure 5:**
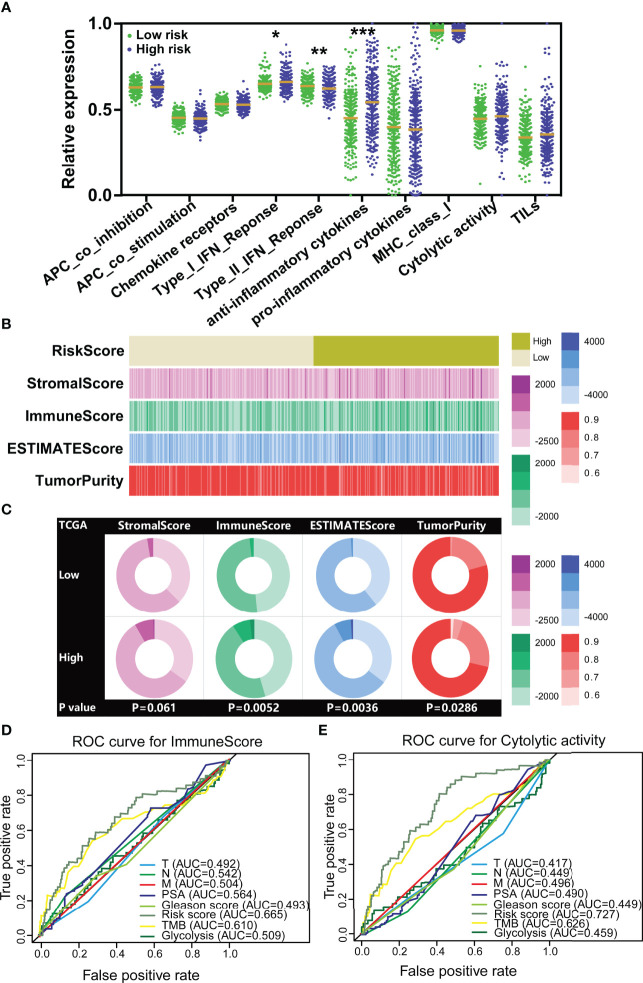
The TILTregSig shows stronger predictive ability for tumor immunity in prostate cancer. **(A)** The expression levels of immune-related signatures in low- and high-risk groups stratified by the TILTregSig in prostate cancer from the TCGA dataset. **(B)** The strip chart of risk score, immune score, stromal score, ESTIMATE score, and tumor purity of prostate cancer patients in the TCGA dataset. **(C)** Pie charts showing the chi-square test of risk score, immune score, stromal score, ESTIMATE score, and tumor purity for low- and high-risk groups in prostate cancer samples from the TCGA dataset. Comparison of the predictive ability of the TILTregSig, TMB, glycolytic activity, PSA levels, Gleason score, Clinical T, Clinical N, and Clinical M for immune score **(D)** and immune cytolytic activity (CYT) **(E)** using ROC curve analysis. *, P<0.05. **, P<0.01. ***, P<0.001.

Because the five genes were initially derived from immune cell lines, we consequently investigated whether the signature was related to the tumor immunity. Therefore, we firstly measured the correlation between the TILTregSig and immune-related factors (chemokines, immunoinhibitors, MHCs, and receptors) and found that the risk score was commonly associated with these immune-related factors. In particular, the TILTregSig has the highest positive correlation with CCL17 followed by CCL14 among 39 chemokines (Spearman correlation: CCL17.R = 0.206, CCL14.R = 0.202, p < 0.0001, **Figure S4**). In 24 immunoinhibitors, the TILTregSig presented the highest positive correlation with LGALS9 followed by TGF-β1 (Spearman correlation: LGALS9.R = 0.218, TGF-β1.R = 0.182, p < 0.0001, **Figure S4**), but showed the highest negative correlation with CD274 followed by TGFBR1 (Spearman correlation: CD274.R = −0,185, TGFBR1.R = −0.175, p < 0.0001, **Figure S4**). Moreover, the TILTregSig has the highest negative correlation with B2M among 21 MHCs (Spearman correlation: R = −0.296, p < 0.0001, **Figure S4**) and CXCL1 among 18 receptors (Spearman correlation: R = −0.244, p < 0.0001, **Figure S4**). Next, further analysis indicated that type I; IFN response and anti-inflammatory cytokines were significantly enhanced in patients with high-risk scores, while type II IFN response was decreased (p < 0.005, **Figure 5A**).

In light of these results, we further conjectured that the TILTregSig was correlated with tumor immunity and might have potential to predict tumor immunity. To test this hypothesis, we firstly employed the ESTIMATE algorithm to quantify the immune score, stromal score, ESTIMATE score, and tumor purity of prostate cancer patients in the TCGA dataset. The results showed that the high-risk group significantly tended to have higher immune scores (p = 0.0052, **Figures 5B, C**) and ESTIMATE score (p = 0.0036, **Figures 5B, C**) and lower tumor purity (p = 0.0286, **Figures 5B, C**) compared to the low-risk group of prostate cancer patients. However, for stromal score, there was no significant difference between low- and high-risk groups (p = 0.061, **Figures 5B, C**). These results illustrate that the TILTregSig is significantly correlated with tumor immunity, which suggests that the TILTregSig promises to predict tumor immunity in prostate cancer.

TMB (35) and glycolytic activity (11) have been demonstrated to have predictive ability for immune signatures. Next, to compare the predictive ability to tumor immunity, we involved TMB and glycolytic signature as reported biomarkers and PSA levels, Gleason score, Clinical T, Clinical N, and Clinical M as classical biomarkers into this analysis. ROC curves indicated that the TILTregSig achieved an AUC of 0.665 in predicting tumor immune score for prostate cancer, while TME achieved an AUC of 0.610 and glycolysis achieved an AUC of 0.509 (**Figure 5D**). The TILTregSig represented moderate potential as an indicator of immune score, as compared to PSA levels, Gleason score, Clinical T, Clinical N, and Clinical M being predictors with minimal potential in prostate cancer (**Figure 5D**). To validate the predictive potential to tumor immunity, we further involved CYT recognized as an immune signature. The TILTregSig achieved an AUC of 0.727 in predicting CYT, while TME achieved an AUC of 0.626 and glycolysis achieved an AUC of 0.459 (**Figure 5E**). For classical biomarkers, PSA levels, Gleason score, Clinical T, Clinical N, and Clinical M showed minimal potential for predicting prostate cancer (**Figure 5E**). Our work strongly indicates that the TILTregSig is significantly correlated with tumor immunity and is a predictor with stronger power for tumor immunity than PSA levels, Gleason score, Clinical T, Clinical N, and Clinical M as classically clinical parameters and some other reported biomarkers in prostate cancer. The TILTregSig is significantly associated with tumor-infiltrating Tregs in prostate cancer.

**Figure 6:**
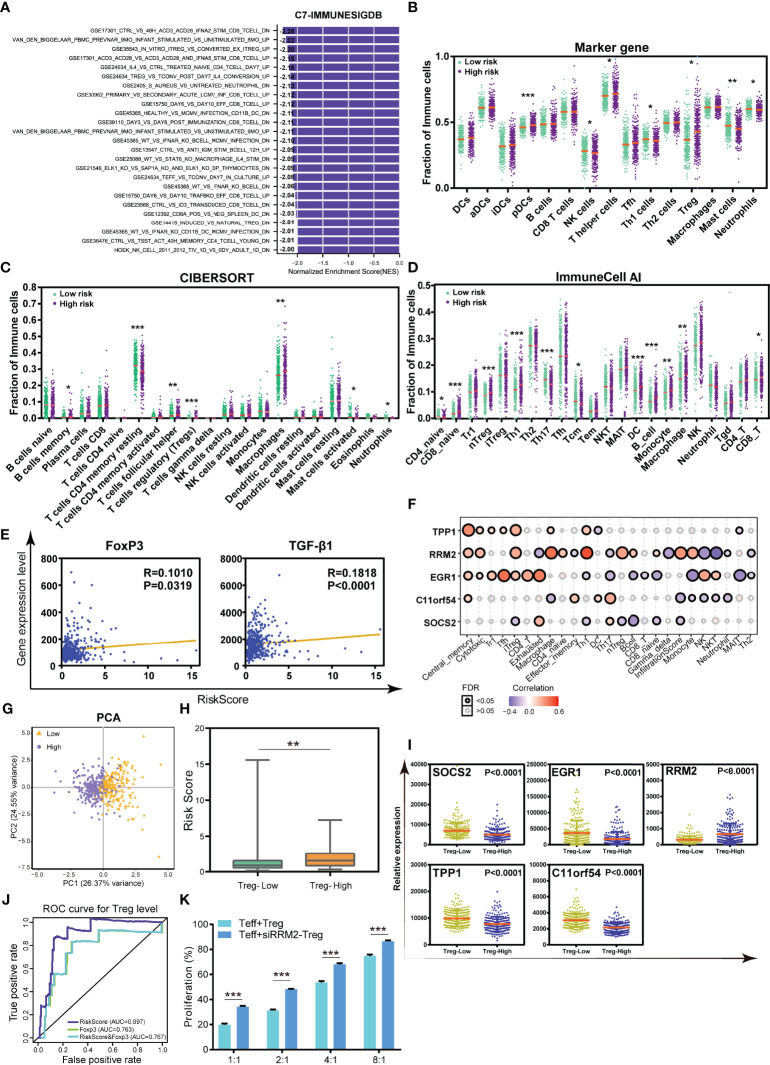
The TILTregSig can serve as a predictor for Tregs in prostate cancer. **(A)** GSEA of the TILTregSig using the gene set of “c7.all.v7.4.symbles.gmt [immunologic signature]”. The infiltration of immune cells in high- and low-risk groups stratified by the risk score in prostate cancer samples from the TCGA dataset using marker genes’ expression analysis **(B)**, CIBERSORT algorithm **(C)**, and ImmuneCell AI database **(D)**. **(E)** The correlation of the TILTregSig with FoxP3 and TGF-β1 expression. **(F)** The correlation of the five genes in the TILTregSig with tumor-infiltrating immune cells in prostate cancer samples from TCGA. **(G)** Principal components analysis (PCA) of the risk scores between Treg-low and Treg-high samples in prostate cancer. The distribution of the risk scores **(H)** and the expression of five genes **(I)** among samples grouped by the infiltration of Tregs in prostate cancer. **(J)** Comparison of predictive ability of the TILTregSig and FoxP3 for Treg infiltration using ROC curve analysis. **(K)** The proliferation rates of Teff cells (Teff:Treg = 1:1, 2:1, 4:1, and 8:1) with normal Tregs or si-RRPM Tregs. *, P<0.05. **, P<0.01. ***, P<0.001.

To elucidate the mechanism of the correlation between the TILTregSig and tumor immunity, we further investigated the immune functional annotation using the gene set of “c7.all.v7.4.symbles.gmt [immunologic signature]” by GSEA. The results demonstrated that the TILTregSig is highly associated with many immune cells, such as CD8+ T cells, CD4+ T cells, B cells, and Tregs (**Figure 6A**). Subsequently, to deeply unveil the relationship between the TILTregSig and tumor-infiltrating immune cells, we then evaluated the infiltration levels of immune cells in high- and low-risk groups in prostate cancer samples using marker genes’ expression analysis, the CIBERSORT algorithm, and the ImmuneCell AI database, respectively. We noticed a consistent result in marker genes’ analysis (**Figure 6B**), the CIBERSORT algorithm (**Figure 6C**), and the ImmuneCell AI database (**Figure 6D**): patients with high-risk scores had a higher infiltration of Tregs compared to those with low-risk scores (Mann–Whitney U test, p < 0.05, **Figures 6B–D**), which suggested a significant correlation between the TILTregSig and Tregs in prostate cancer. To validate these results, we further involved Tregs’ marker genes (FoxP3 and TGF-β1) into our study, and the correlation analysis demonstrated that the TILTregSig was significantly positively related to FoxP3 and TGF-β1 expression (p < 0.05, **Figure 6E**), which was consistent with our previous results (**Figures 6B–D**). Additionally, RRM2 showed the highest correlation with iTreg cells and nTreg cells among these five genes in the TILTregSig (**Figure 6F**), suggesting that RRM2 might be a key gene to determine the correlation between the TILTregSig and tumor immunity. These results suggest that the TILTregSig may influence tumor immunity mainly by mediating tumor-infiltrating Tregs, and RRM2 may play a vital role in this section, which needs to be verified by further experiments.

### The TILTregSig Is a Powerful Predictor for the Infiltration of Tregs in Prostate Cancer

In view of the relationships between Tregs and the TILTregSig in the TME, we further conjectured that the TILTregSig had potential for predicting the infiltration of Tregs in prostate cancer. To test this hypothesis, prostate cancer patients in the TCGA dataset were firstly split into the Treg-low group (n = 227) and the Treg-high group (n = 227) based on the median infiltration levels of Tregs. Principal component analysis (PCA) demonstrated that the TILTregSig was able to distinguish Treg-low cluster from Treg-high cluster (**Figure 6G**), which preliminary hinted to us that the TILTregSig had the potential of predicting the infiltration of Tregs in prostate cancer.

Additionally, we found that patients in the Treg-high group had significantly higher risk scores compared to those in Treg-low group (p < 0.001, **Figure 6H**). In particular, the expression of SOCS2, EGR1, TPP1, and C11orf54 was markedly lower in patients with high infiltration levels of Tregs compared to patients with low infiltration levels of Tregs, while RRM2 was markedly higher (p < 0.0001, **Figure 6I**). These results reconfirmed the predictive potential of the TILTregSig for Tregs’ infiltrations in prostate cancer.

Therefore, to evaluate the predictive power of the TILTregSig for Tregs’ infiltrations, ROC curves were employed in further analyses. Additionally, FoxP3 has been proven as a classic indicator of Tregs (36). Therefore, we also involved FoxP3 in this analysis to compare the predictive ability to the TILTregSig. Surprisingly, we found that the TILTregSig represented high potential as an indicator of Tregs (AUC = 0.897, **Figure 6J**), as compared to FoxP3 being a predictor with moderate potential in prostate cancer (AUC = 0.763, **Figure 6J**). Moreover, we further constructed a combined model consisting of the TILTregSig and FoxP3, and found that the combined model had weaker predictive power compared with the TILTregSig (AUC = 0.767, **Figure 6J**). These results suggest that the TILTregSig is a robust and accurate predictor for Tregs in prostate cancer, and its predictive power is stronger than FoxP3.

### RRM2 Downregulation Attenuates the Suppressive Function of Tregs

In order to investigate the influence of RRM2 as a risk factor in our TILTregSig on Treg function, Tregs were treated with small interfering RNA (siRNA) to knock down RRM2 expression and then si-RRPM Tregs were stimulated and cultured with CD4+ CD25- T cells (Teff) as described in Methods. CCK8 assay was employed to detect the suppressive function of Tregs. The proliferation rates of Teff cells (Teff:Treg = 1:1, 2:1, 4:1, and 8:1) with normal Tregs or si-RRPM Tregs were compared. Subsequently, we showed that RRM2 knockdown led to an enhancement of the proliferation rate of Teff cells, which suggested that RRM2 might contribute to the suppressive function of Tregs (**Figure 6K**).

### The TILTregSig Has Predictive Potential as an Indicator of Response to CIT

**Figure 7:**
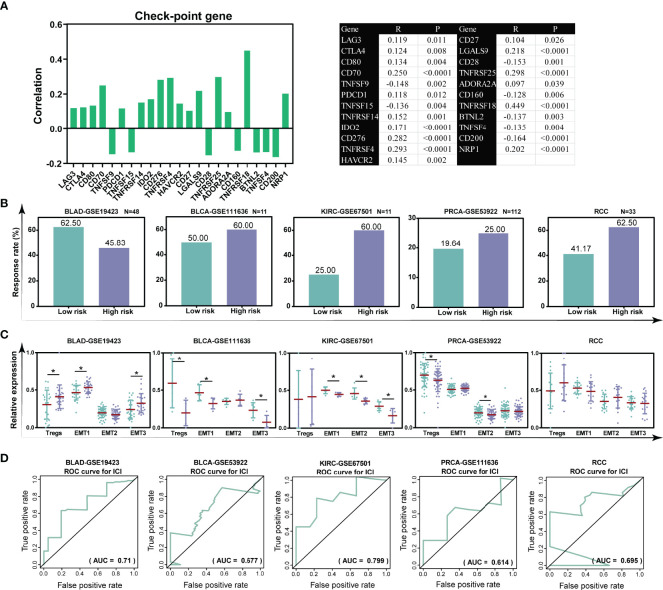
The TILTregSig is a potential predictor for immunotherapy response in prostate cancer. **(A)** The correlation of the risk scores with the expression levels of checkpoint genes in prostate cancer samples from the TCGA dataset. *R*: Spearman’s correlation coefficient. **(B)** The response rate to immunotherapy in low- and high-risk groups stratified by risk scores in each dataset. **(C)** The distribution of the Treg infiltration and EMT activity among samples in low- and high-risk groups in each dataset. **(D)** ROC curves of the TILTregSig in predicting immunotherapy response in each dataset. *, P<0.05.

Accumulating evidence demonstrated that patients with a low infiltration of Tregs presented a durable clinical response to CIT (37). Our previous data demonstrated that the TILTregSig was associated with Tregs, suggesting that the TILTregSig might be a pivotal factor that mediated the clinical response to CIT. The correlation between our TILTregSig and checkpoint genes’ expression indicated that the risk score was markedly correlated with checkpoint genes’ expression, and most were positively correlated (p < 0.05, **Figure 7A**). Subsequently, to investigate whether the TILTregSig could predict patients’ response to CIT, we utilized five CIT response-associated datasets (GSE19423, GSE111636, GSE67501, GSE53922, and Miao et al. (25)). We found that the significant therapeutic advantages and clinical response to CIT in patients with high-risk score compared to those with low-risk score were confirmed in bladder urothelial carcinoma (BLCA), kidney renal clear cell carcinoma (KIRC), prostate carcinoma (PRCA), and renal cell carcinoma (RCC). However, in bladder adenocarcinoma (BLAD), patients with high-risk scores had lower immunotherapy response rate compared to those with low-risk scores (**Figure 7B**). Tregs and TME stroma activity, which usually mediated the immune tolerance of tumors, was also assessed (12, 38). We found that Tregs were significantly activated in tumors with high-risk scores in the BLAD-GSE19423 dataset, but were inhibited in BLCA-GSE111636 and PRCA-GSE53922 datasets (p < 0.05, **Figure 7C**). Additionally, TME stroma activity was significantly enhanced in patients with high-risk scores such as the activation of EMT in the BLAD-GSE19423 dataset, but was decreased in tumors with high-risk scores in the BLCA-GSE111636 and PRCA-GSE53922 datasets (p < 0.05, **Figure 7C**). The above data imply that the TILTregSig is correlated with CIT response and might have potential for predicting CIT response. To assess the predictive power of the TILTregSig for CIT response, ROC curves were employed in this work. The results showed that the TILTregSig achieved AUCs of 0.710, 0.677, 0.799, 0.614, and 0.695 for BLAD-GSE19423, BLCA-GSE111636, KIRC-GSE67501, PRCA-GSE53922, and RCC, respectively, in predicting the response to CIT, which implied that the TILTregSig was a potential and robust biomarker for response assessment of CIT with moderate predictive potential (**Figure 7D**). In summary, our work strongly indicated that the TILTregSig was significantly correlated with immunotherapy response, and the established TILTregSig would contribute to predicting the response to CIT.

### The TILTregSig Is a Promising Marker of Therapeutic Resistance in Pan-Cancers

**Figure 8:**
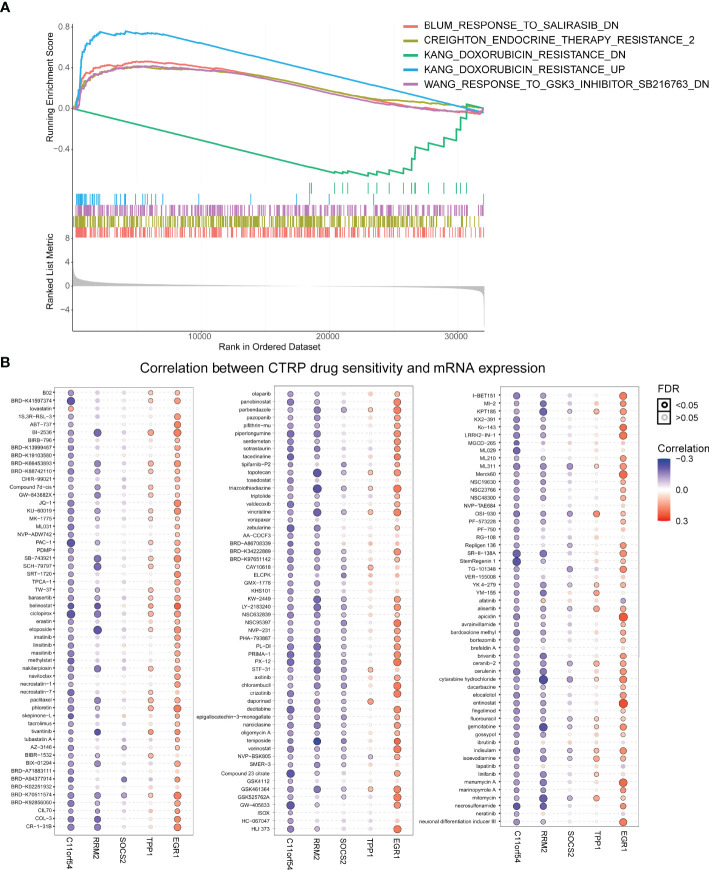
The TILTregSig is a promising marker of cancer therapeutic resistance. **(A)** The relationship between the TILTregSig and therapeutic resistance using GSEA. **(B)** A landscape plot was generated to depict the relationships between the IC_50_ data of different molecules and the five genes’ expression profiles in cancers using GSCALite.

Considering Treg cells have been indicated to usually promote resistance to cancer therapy (39), we investigated whether our TILTregSig was correlated with cancer therapeutic resistance. To unveil the relationship between the TILTregSig and therapeutic resistance, we utilized GSEA. As shown in **Figure 8A**, GSEA predicted that the TILTregSig was significantly associated with resistance to different therapies, including salirasib, endocrine therapy, doxorubicin, and SB216763 in prostate cancer (p < 0.05, **Figure 8A**). Next, a landscape plot was generated by GSCALite to depict the relationships between the five genes’ expression in the TILTregSig and drug responses in pan-cancers. The bubble heat map showed that some genes exhibited significant correlations with lower half-inhibitory concentration (IC50) data. In detail, EGR1, TPP1, and SOCS2 conferred drug resistance, while RRM2 and C11orf54 exhibited drug sensitivity in pan-cancers (**Figure 8B**). These results imply that the TILTregSig is a promising indicator for therapeutic resistance in pan-cancers.

## Discussion

TILs serve as the cellular underpinnings of cancer immunotherapies, and a better understanding of TILs in the TME is essential for deciphering mechanisms of immunotherapies, defining predictive biomarkers, and identifying novel therapeutic targets. As a key component of the TILs, Tregs usually play a pivotal role in tumor development and progression due to their immunosuppressive functions in the TME. Therefore, better benefits of improving survival of cancer patients can be realized if Tregs can be quantitatively evaluated. However, the traditional method of TIL quantification, including making visual measurements through a microscope by pathologists using hematoxylin and eosin- or immunohistochemistry-stained tumor sections, usually suffers from bias and variability (40). Therefore, identification of a Treg-specific signature based on genomic profiles may open up a new path to the prediction for survival and immunotherapy response of cancer patients.

In our study, we develop and validate a prognostic signature (named TILTregSig) composed of five Treg-specific mRNAs (SOCS2, EGR1, RRM2, TPP1, and C11orf54) for prostate cancer, which is associated with RFS in prostate cancer. Moreover, our data further show a significantly positive correlation between the TILTregSig and stemness indices in both transcriptomic (mRNA expression) and epigenetic (DNA methylation) levels. These observations consistently indicate that the TILTregSig is a risk factor for prognosis of prostate cancer.

Suppressors of cytokine signaling (SOCS) has been identified as an inhibitor for prostate cancer progression (41, 42). Downregulation of SOCS2 was an independent predictor of shorter biochemical recurrence-free survival for prostate cancer patients (43). Early growth response-1 (EGR1) is a transcription factor involved in cell proliferation and in the regulation of apoptosis. Several previous studies showed that EGR1 exhibits prominent tumor suppressor function in glioma (44), non-small-cell lung cancer (45), colon cancer (46), papillary thyroid carcinoma (47), and breast cancer (48). Saha et al. indicated that low levels of EGR1 expression were positively correlated with poor survival for RFS and distant metastasis-free survival (DMFS) in breast cancer (49). Ribonucleotide reductase small subunit M2 (RRM2), as a master driver of aggressive prostate cancer (50), showed significant prognostic value of RRM2 in prostate cancer (51). These studies are consistent with our results. Liu et al. reported that the telomere shelterin protein TPP1 can activate telomerase for telomere repeat synthesis (52). C11orf54 was found to be downregulated in clear cell renal cell carcinoma and might be a potential biomarker for the diagnosis of clear cell renal cell carcinoma (53). However, there has been no study that reported the potential function of TPP1 or C11orf54 in prostate cancer. Our study might fill this gap and lays the foundation for future experimental exploration of the potential roles of these genes in prostate cancer.

Cancer is a disease driven by genetic variation and mutation (33), and prostate cancer has been recognized as having high intratumoral genetic heterogeneity (54). We also identify the landscape of genetic variations of the five genes in TILTregSig in prostate cancer. The results demonstrate that the EGR1 exhibited the highest mutation frequency followed by RRM2 and TPP1, while both SOCS2 and C11or54 do not show any mutations in prostate cancer samples. Moreover, the high expression of SOCS2 and TPP1 is prone to have a high CNV load. A previous study indicated that CNV and SNV status was reported to be significantly associated with overall cancer risk and metastasis (54–56). These data suggest genetic alterations as the molecular mechanism that shows that high-risk score is related to poor prognosis in prostate cancer.

In addition, our data show that type I; IFN response and anti-inflammatory cytokines are significantly enhanced in prostate cancer patients with high-risk scores. A large body of evidence indicated that type I; IFN response was emerging as a key driver of immunosuppression and tumor progression (57). Anti-inflammatory cytokines are proved to be usually involved in cancer progression and related with worse prognosis (58). These previous studies are consistent with our findings that prostate cancer patients with high-risk scores have high levels of type I; IFN response and anti-inflammatory cytokines, and are related to poor prognosis. This observation has important implications for comprehending the mechanism of the influence of the TILTregSig on anti-tumor immunity.

Moreover, we also reveal that the TILTregSig significantly correlates with tumor immunity. TMB (7) and glycolytic activity (11) have been demonstrated to have promising potential to predict tumor immunity. Subsequently, in comparison with TMB and glycolytic activity, the TILTregSig displays higher predictive power for predicting tumor immunity than TMB and glycolytic activity, with moderate predictive potential. This observation enhances the predictive accuracy of the evaluation of tumor immunity based on existing markers in prostate cancer, which have tremendous significance to improve the prognosis of prostate cancer patients.

Furthermore, the TILTregSig shows higher potential as an indicator of Tregs in prostate cancer with moderate potential, while it is recognized as a hallmark of Tregs compared to FoxP3 (36). A high infiltration of Tregs is associated with poor survival in various cancers (59). Tregs act on innate immune cells and effector T cells to suppress the anticancer immunity that is mediated by natural killer cells, cytotoxic CD8+ T cells, and pro-inflammatory cytokines through secretion of inhibitory cytokines, such as IL-10, TGF-β, and IL-35 (18, 60, 61). Moreover, IFN-Is can also enhance the suppressive effects of Tregs, and the IFN-I production in the tumor drives the suppressive Tregs’ phenotype (62). Our previous results show that type I; IFN response and anti-inflammatory cytokines are significantly enhanced in prostate cancer patients with high-risk scores. Based on the above data, we infer that Tregs may affect prostate cancer prognosis through exerting their immunosuppressive functions by regulating type I; IFN response and the secretion of anti-inflammatory cytokines.

CIT is a validated and critically important approach for treating patients with cancer. A large body of evidence indicated that Tregs were prevalent in nearly all cancers and, as immunosuppressive regulators of immune responses, they were strongly associated with the response of CIT (15, 17). Given the correlation between the TILTregSig and Tregs, we involve five CIT response-associated datasets [GSE19423, GSE111636, GSE67501, GSE53922, and Miao et al. (25)], and find that the TILTregSig is a promising biomarker for predicting CIT response. Further investigation is therefore warranted to establish the potential utility of the TILTregSig as an additional measure to identify patients likely to respond to CIT. In addition, these findings support the development of agents targeting tumor-infiltrating Tregs for use in combination with existing CIT. However, using an agent that targets Tregs considered to be predictive of response to this class of agents requires further clinical validation. As our signature has been derived independently of any specific molecular agent targeting the tumor-infiltrating Tregs, it may have widespread utility of candidate drugs currently in development.

Given that Tregs have been indicated to usually promote resistance to therapy (39), we reason that the TILTregSig can be applicable to the prediction of cancer therapeutic resistance. We firstly find that the TILTregSig is significantly associated with resistance to different therapies, including salirasib, endocrine therapy, doxorubicin, and SB216763 in prostate cancer. Further analysis reveal that EGR1, TPP1, and SOCS2 confer drug resistance, while RRM2 and C11orf54 exhibit drug sensitivity in pan-cancers. A previous study demonstrated that EGR1 is related to cancer therapy resistance, such as radiation resistance (63) and drug resistance (64). SOCS2 was also proved as a therapeutic resistance-related gene for cancers (65). Altogether, these results indicate that the TILTregSig can also serve as a promising marker for therapeutic resistance in cancers. Of course, the association between the TILTregSig and therapeutic resistance revealed in this study needs to be validated in a clinical setting.

Our results presented one unexpected finding: patients with BLCA, KIRC, PRCA, and RCC in high-risk groups were prone to have a better response to CIT, while patients with high-risk scores had a lower CIT response rate in BLAD. Tregs and TME stroma activity, such as EMT activity, usually induce tumor immune tolerance and relate to low CIT response rate (12, 38). Further investigation shows that Tregs are significantly activated in tumors with high-risk scores in BLAD, but are inhibited in BLCA and PRCA. Additionally, EMT activity is significantly enhanced in patients with high-risk scores in BLAD, but is decreased in BLCA, KIRC, and PRCA. Given these data, we confer that the distinction of CIT response rate in diverse cancers may be due to the different activations of Tregs and EMT in diverse cancers.

Despite the significant results obtained in the current study, there are inevitably several shortcomings of our study that should be acknowledged. First, the signature described here may be limited by the decision to only include genes within publicly available datasets, which may introduce bias into the results. Second, transcriptomics analysis can reflect only some aspects of immune status rather than global alterations. Third, the reliability of our results from the bioinformatics analysis is still challenged by the lack of *in vitro* or *in vivo* experiments.

## Conclusion

In conclusion, in this work, a machine learning-based computational framework based on immune, mRNA, and clinical profiles has been presented for identifying a Treg-specific prognostic signature (TILTregSig) for patients with prostate cancer. The TILTregSig displays an independently predictive potential for the prognosis of prostate cancer patients, even when adjusting for clinical covariates. Further analyses indicate that the TILTregSig may influence tumor immunity mainly by mediating tumor-infiltrating Tregs, and it can serve as a potential indicator for tumor-infiltrating Tregs in prostate cancer. Moreover, we also find that the TILTregSig is capable of assessing CIT response in multiple cancers and shows a promising potential for predicting cancer therapeutic resistance. Our TILTregSig derived from PBMCs makes it possible to achieve a straightforward, noninvasive, and inexpensive detection assay for prostate cancer compared with the current histopathological examination that requires invasive tissue puncture, which lays the foundation for the future development of a panel of different molecules in peripheral blood comprising a biomarker of prostate cancer.

## Data Availability Statement

The datasets presented in this study can be found in online repositories. The names of the repository/repositories and accession number(s) can be found in the article/**Supplementary Material**.

## Author Contributions

Conceptualization: MJ. Collection and Assembly of Data: JB, LJ, and QW. Data Analysis and Interpretation: YW, BH, LW, and QG. Original Draft Preparation: XS, LY, and MD. Writing: JF, YZ, and MY. Visualization and Supervision: HK, WX, and LZ. All authors contributed to the article and approved the submitted version.

## Funding

This work was supported by grants from the National Natural Science Foundation of China (Nos. 82073281, 82073884, U20A20413, and 81903658), the Science and Technology Program of Liaoning Province (2017225036), Shenyang S&T Projects (Nos. 19-109-4-09 and 20-204-4-22), the Program for Shenyang High Level Talent Innovation and Entrepreneurship Team (2019-SYRCCY-B-01), the Liaoning Revitalization Talents Program (No. XLYC1807201), and Major Special S&T Projects in Liaoning Province [2019JH1/10300005].

## Conflict of Interest

The authors declare that the research was conducted in the absence of any commercial or financial relationships that could be construed as a potential conflict of interest.

## Publisher’s Note

All claims expressed in this article are solely those of the authors and do not necessarily represent those of their affiliated organizations, or those of the publisher, the editors and the reviewers. Any product that may be evaluated in this article, or claim that may be made by its manufacturer, is not guaranteed or endorsed by the publisher.

## Supplementary Material

Supplementary Figure 1
**(A)** CD4+CD25+CD127- Tregs and **(B)** CD4+CD25- T cells (Teff) were enriched using flow cytometry. Antibodies specific for CD4, CD25 and CD127 were used to distinguish viable target cells.

Supplementary Figure 2 The correlations of the TILTregSig with cancer stem scores in the RNA expression levels **(A)** and DNA methylation levels **(B)**.

Supplementary Figure 3 The TILTregSig is an independent prognostic factor for prostate cancer patients. Univariate **(A)** and multivariate **(B)** Cox regression of prognosis factor for RFS of prostate cancer patients. **(C)** Kaplan–Meier analysis of RFS for prostate cancer patients stratified by stage T, N, age, race, treatment response, postoperative RX and laterality.

Supplementary Figure 4 The correlation between the TILTregSig and immune-related factors, including chemokines, immunoinhibitors, MHCs and receptors.
