# Supplementary figures and images for: Computational Recognition of a Regulatory T-cell-specific Signature With Potential Implications in Prognosis, Immunotherapy, and Therapeutic Resistance of Prostate Cancer
Source: Front Immunol. 2022 Jun 23;13:807840. doi: 10.3389/fimmu.2022.807840 (PMC9259848; doi:10.3389/fimmu.2022.807840)

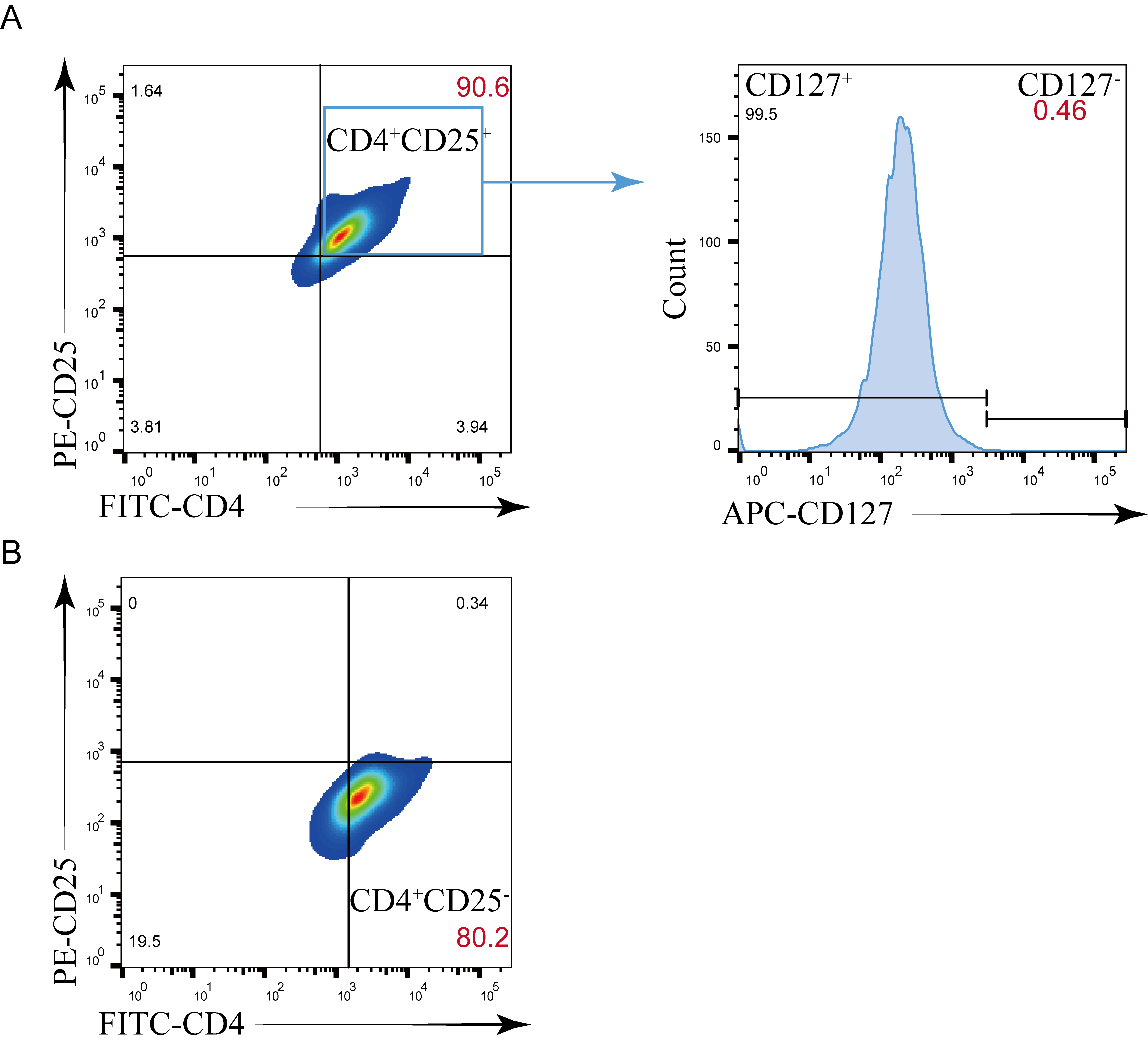

Supplement: Supplementary Figure 1 — (A) CD4+CD25+CD127- Tregs and (B) CD4+CD25- T cells (Teff) were enriched using flow cytometry. Antibodies specific for CD4, CD25 and CD127 were used to distinguish viable target cells. [file Image_1.tif]

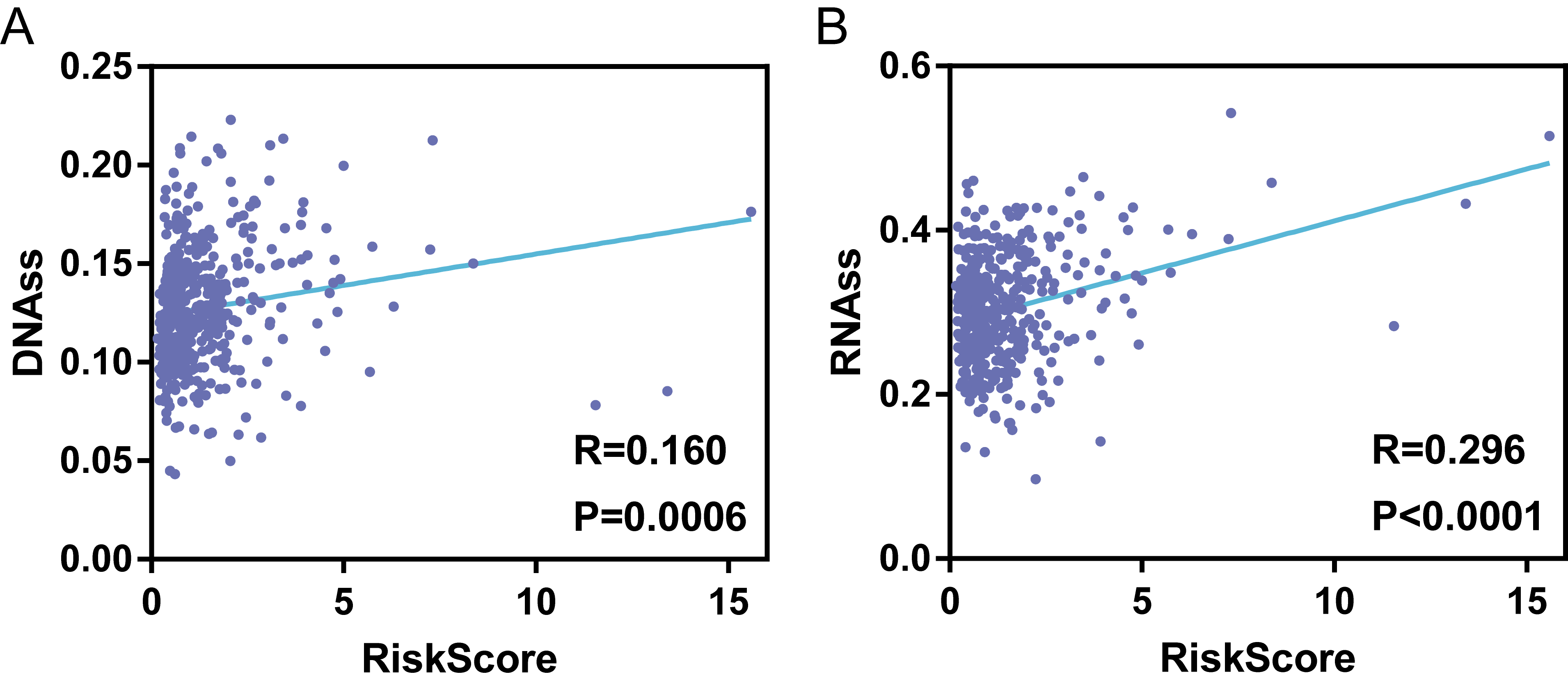

Supplement: Supplementary Figure 2 — The correlations of the TILTregSig with cancer stem scores in the RNA expression levels (A) and DNA methylation levels (B). [file Image_2.tif]

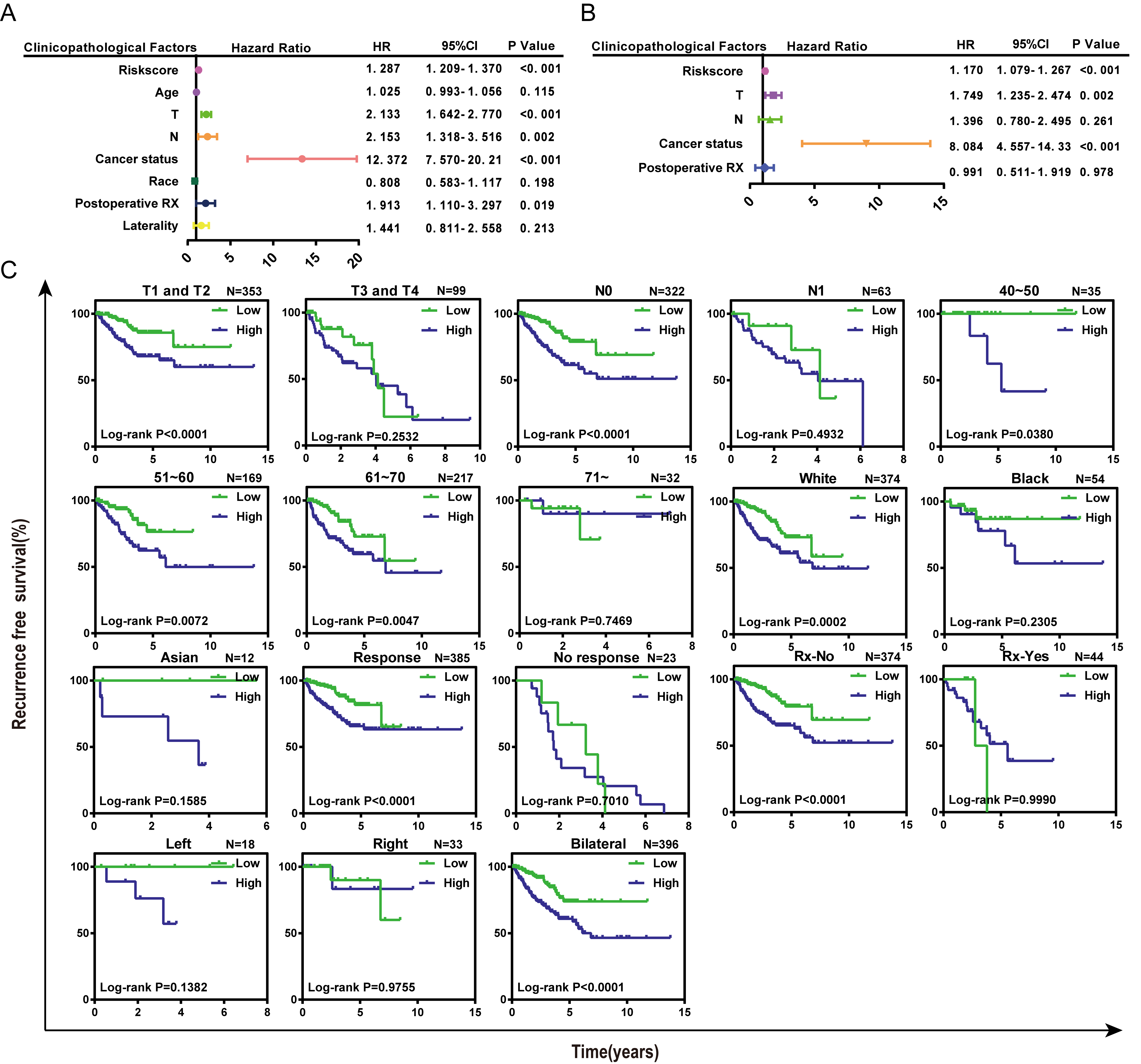

Supplement: Supplementary Figure 3 — The TILTregSig is an independent prognostic factor for prostate cancer patients. Univariate (A) and multivariate (B) Cox regression of prognosis factor for RFS of prostate cancer patients. (C) Kaplan–Meier analysis of RFS for prostate cancer patients stratified by stage T, N, age, race, treatment response, postoperative RX and laterality. [file Image_3.tif]

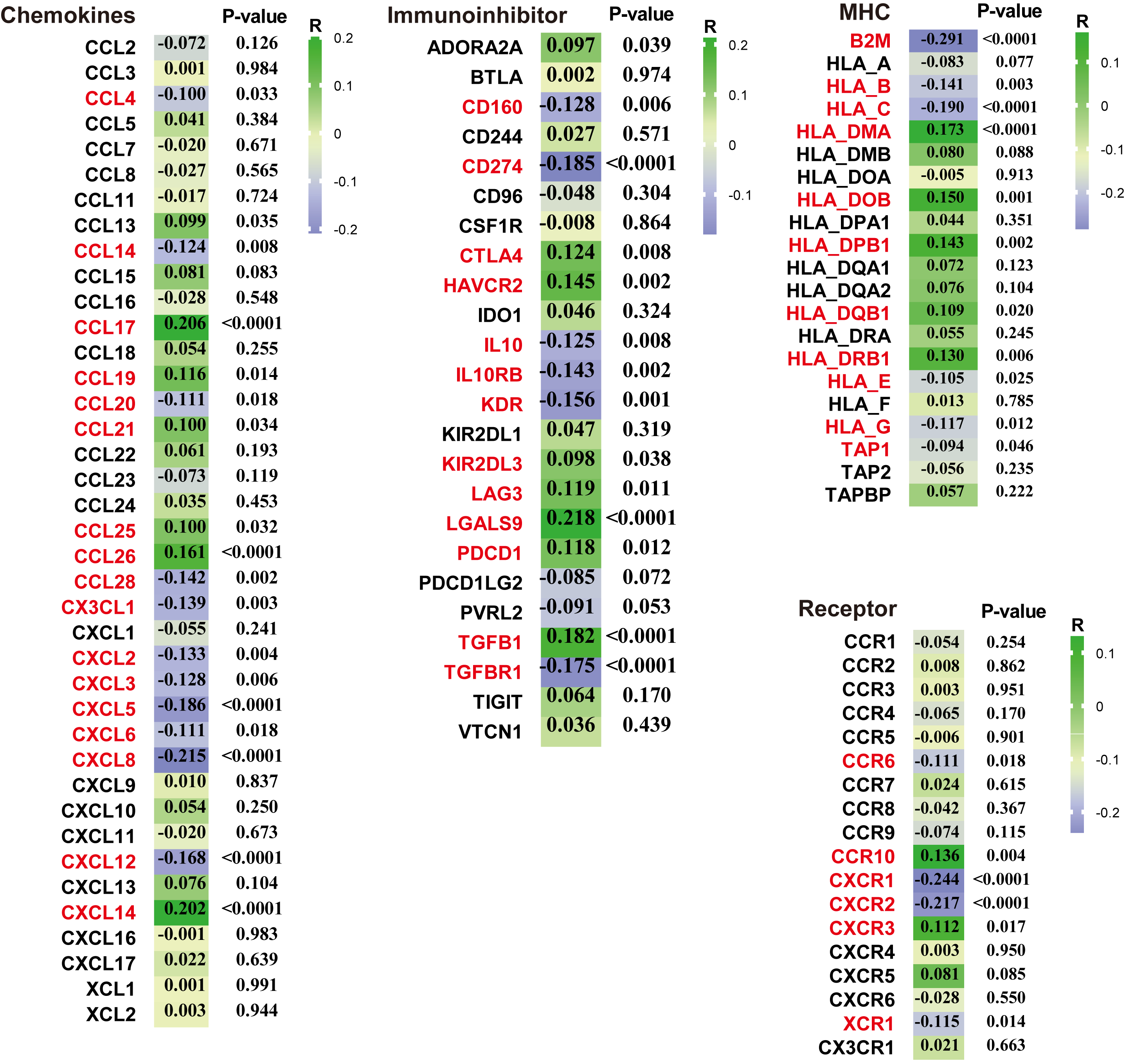

Supplement: Supplementary Figure 4 — The correlation between the TILTregSig and immune-related factors, including chemokines, immunoinhibitors, MHCs and receptors. [file Image_4.tif]
